# Assessing the knowledge and skills on emergency obstetric care among health providers: Implications for health systems strengthening in Nigeria

**DOI:** 10.1371/journal.pone.0213719

**Published:** 2019-04-08

**Authors:** Friday Okonofua, Lorretta Favour Chizomam Ntoimo, Rosemary Ogu, Hadiza Galadanci, Mohammed Gana, Durodola Adetoye, Eghe Abe, Ola Okike, Kingsley Agholor, Rukiyat Adeola Abdus-salam, Abdullahi Randawa, Hauwa Abdullahi, Suleiman Muhammad Daneji, Blessing Itohan Omo-Omorodion

**Affiliations:** 1 The Women’s Health and Action Research Centre/WHO Implementation Research Group, Benin, Nigeria; 2 University of Medical Sciences, Ondo, Ondo, Nigeria; 3 Centre of Excellence in Reproductive Health Innovation, University of Benin, Benin, Edo, Nigeria; 4 Department of Demography and Social Statistics, Federal University Oye-Ekiti, Oye-Ekiti, Ekiti, Nigeria; 5 Department of Obstetrics and Gynaecology, University of Port Harcourt, Port Harcourt, Rivers, Nigeria; 6 Aminu Kano Teaching Hospital, Kano, Kano, Nigeria; 7 General Hospital, Minna, Niger, Nigeria; 8 General Hospital, Ijaye Abeokuta, Ogun, Nigeria; 9 Central Hospital, Benin, Edo, Nigeria; 10 Karshi General Hospital, Federal Capital Territory, Abuja, Nigeria; 11 Central Hospital, Warri, Delta, Nigeria; 12 Adeoyo Maternity Hospital, Ibadan, Oyo, Nigeria; 13 Ahmadu Bello University Teaching Hospital, Zaria, Kaduna, Nigeria; The University of Warwick, UNITED KINGDOM

## Abstract

**Objective:**

To assess the existing knowledge and skills relating to Emergency Obstetrics Care (EMOC) among health providers in eight referral maternity hospitals in Nigeria.

**Study design:**

A cross-sectional study of skilled health providers (doctors, nurses and midwives) working in the hospitals during the period.

**Setting:**

Six general hospitals (4 in the south and 2 in the north), and two teaching hospitals (both in the Northern part) of the country.

**Population:**

All skilled providers offering EMOC services in the hospitals during the study.

**Methods:**

A pre-tested self-administered questionnaire was used to obtain information relating to socio-demographic characteristics, the respondents’ knowledge and skills in offering specific EMOC services (as compared to standard World Health Organization recommendations), and their confidence in transferring the skills to mid-level providers. Data were analyzed with univariate, bivariate, binary and multinomial logistic regression analyses. Main outcome measures: knowledge and skills in EMOC services by hospital and overall.

**Results:**

A total of 341 health providers (148 doctors and 193 nurses/midwives) participated in the study. Averagely, the providers scored less than 46% in a composite EMOC knowledge score, with doctors scoring considerable higher than the nurses/midwives. Similarly, doctors scored higher than nurses/midwives in the self-reporting of confidence in carrying out specific EMOC functions. Health providers that scored higher in knowledge were significantly more likely to report confidence in performing specific EMOC functions as compared to those with lower scores. The self-reporting of confidence in transferring clinical skills was also higher in those with higher EMOC knowledge scores.

**Conclusion:**

The knowledge and reported skills on EMOC by health providers in referral facilities in Nigeria was lower than average. We conclude that the in-service training and re-training of health providers should be included in national policy and programs that address maternal mortality prevention in referral facilities in the country.

**Trial registration:**

Nigeria Clinical Trials Registry 91540209.

## Introduction

Available evidence indicate that while 303,000 women died in 2015 from preventable causes related to pregnancy and childbirth, sub-Saharan Africa accounted for 66% of these deaths [1]. Within sub-Saharan Africa, nearly 29% of the maternal deaths occurred in Nigeria with an estimated 58,000 pregnant women dying annually in the country mainly from preventable obstetric causes [1, 2]. While many socio-economic and cultural factors have been put forward to explain the high rates of maternal deaths in Nigeria [3, 4], it is evident that the poor accessibility of pregnant women to quality emergency obstetric care (EMOC) is at the root of the problem. The WHO defines EMOC as “a list of live-saving services or signal functions that define a health facility with regard to its capacity to treat obstetric emergencies” [5]. Two types of EMOC services have been described: Basic EMOC (BEOC) and Comprehensive Emergency Obstetrics Services (CEOC). BEOC is offered in primary health care facilities and consists of skilled delivery care, the administration of antibiotics, manual removal of the placenta, removal of retained products of conception, assisted vaginal delivery possibly with a vacuum extractor, and basic neonatal care including neonatal resuscitation. In contrast, CEOC consists of all BEOC services as well as caesarean section, safe blood transfusion services and the treatment of the sick baby, and is offered mainly in referral Secondary and Teaching Hospitals by skilled medical and midwifery personnel. Although the Federal Ministry of Health of Nigeria recommends BEOC as the entry point to the health care system in order to generate universal health coverage for all citizens [6], it is evident that a large number of women require CEOC because of the complexity of their pregnancy complications.

Several reports indicate that a large number of women tend not to attend basic antenatal care or delivery services in health facilities, but rather attempt to deliver at home or with traditional or faith-based birth attendants. As such, when women experience severe complications of pregnancy, they are brought to referral secondary or tertiary health care facilities for CEOC, sometimes in very dire conditions [4, 7, 8]. To date, the poor organization of CEOC in Nigeria has been well documented [8]. Apart from the lack of facilities and organizational preparedness [4, 9] for CEOC, there is also the inadequate knowledge and skills of health providers in delivering CEOC in line with current international standards. Our early study showed that up to 40 percent of maternal deaths occurring in a teaching hospital were attributable to inadequacies in the health care system, including the inappropriate delivery of EMOC by health personnel [10, 11]. Additional reports from several referral facilities in the country and elsewhere in sub-Saharan Africa [12, 13, 14] have also reported high case-fatality rates as well as high rates of maternal mortality in secondary and tertiary hospitals due to poor quality obstetric services.

While inadequate and poor EMOC services may explain the high case fatality associated with obstetric complications that reach referral hospitals, inadequate knowledge and poor skills of providers in the provision of related services is also part of the problem. Previous studies have investigated the knowledge and skills of health providers in sub-Saharan Africa in providing EMOC. A systematic review of the literature by Fikre [15] concluded that poor skills of health providers and the absence of essential equipment and shortages of health providers account for the inadequate delivery of EMOC in developing countries. A similar review by Jonas *et al*. [16] reported inadequate knowledge of EMOC among health providers in sub-Saharan Africa and emphasized that the required skills are insufficient to provide high quality EMOC.

Similar studies have been reported from Nigeria where severe shortages have been identified in health workers’ knowledge and practices of EMOC, especially in rural areas as compared to urban areas [17].

A major approach to solve this bottleneck has included the provision of competency-based training and efforts devoted to promoting the retention of practical knowledge and skills by health workers [18, 19, 20]. While these interventions have proven to be effective at the local institutional levels, no efforts have been made to integrate the continuous training and re-training of health workers on EMOC practices to policies and practices at the health systems level. It is evident that trainings conducted without reference to the healthcare system will have limited chance of being effective at scale over the long term.

We conducted a study to assess the knowledge, skills and practices of health providers on emergency obstetrics care in eight referral hospitals in four out of the six geo-political zones of Nigeria. The objective of the study was to identify gaps in knowledge and skills relating to EMOC that would enable the design of policies and programs for integrating the sustained capacity building of health providers on emergency obstetric care. EMOC being an increasingly innovative program for which new methods and practices are thrown up on a regular basis, it is important that managers of the healthcare system develop a strategy for bringing such new methods to the knowledge of health providers. We believe that this approach stands a better chance of sustainable success in improving the knowledge and skills of health providers to offer EMOC in low income countries.

## Methods

### Study sites and health institutions

The data were obtained from a formative research designed to assess the nature and quality of EMOC to prevent maternal and perinatal mortality in eight secondary and tertiary hospitals in Nigeria. Nigeria’s EMOC is founded on a two-tier system: BEOC at the Primary Health Care (PHC) as the entry point; and CEOC at Secondary Care (regional/general hospitals/teaching hospitals) as the referral and more comprehensive levels of care for women requiring more sophisticated methods of care.

For this study, eight referral hospitals (two teaching and six General hospitals) were randomly selected from four out of the six geo-political zones of Nigeria. Administratively, Nigeria has 36 states and a Federal Capital Territory (Abuja). The states are further categorized into six zones: North-central, Northeast, Northwest, Southeast, South-south, and Southwest, with each zone made up predominantly of people of similar culture. Two tertiary hospitals were selected from the Northwest: Ahmadu Bello University Teaching Hospital, Kaduna (ABUTH) and Aminu Kano Teaching Hospital Kano (AKTH). By contrast, the secondary care facilities were selected from three other zones: General Hospital, Ijaye, Abeokuta (SHI) and Adeoyo Maternity Hospital (AMHI) in the Southwest; General Hospital, Minna (GHM), Niger State and Karshi Hospital (KGH), Abuja in the North-central, and Central Hospital, Benin City (CHB) and Central Hospital, Warri (CHW) from the South South. We did not select any referral hospital from the North-east zone because of the difficulty in reaching the hospitals as a result of the ongoing insurgency in the zone. To equalize the selection, we also did not sample hospitals in the contiguous south-east zone. The hospitals were selected on the basis that they are the main referral hospitals in the respective states; they attend to large populations of pregnant women in the main cities of the States; with none having less than 4,500 total deliveries per year.

Furthermore, a large proportion of women often present in labour in the hospitals as obstetric emergencies who had intended to deliver at home, in private clinics or in the homes of traditional birth/faith-based birth attendants, but then come late to the hospitals when they experience complications. Such deliveries often constitute about 50% of cases seen in the hospitals, accounting for an average of about 150 obstetric emergencies per month per hospital. All the hospitals are funded either by the respective States or the Federal Government, which means that policies on staffing of the hospitals are also overseen by government departments.

Since this was a formative study leading to the design of a larger intervention, we decided to choose pairs of states with catchment populations of similar socio-demographic characteristics that will adequately represent the research design. We first choose the largest referral hospitals in the four states. Based on our knowledge of use of maternal health services in the states, we considered that General hospitals have larger uptake of obstetric emergencies, have fewer health providers, and that health providers in General Hospitals may have less opportunity for continued in-service training. For this reason, we decided to sample three General hospitals as compared to only one teaching hospital. In the second stage of sampling we chose comparative main referral hospitals in adjoining states different from the states where the initial selections were made. This was to eliminate the effect of intervention contamination, should we implement the intervention at a later stage. In particular, we decided to choose teaching hospitals from the northern part of the country, because of the reported higher numbers of deliveries and maternal deaths in those northern Nigeria hospitals as compared to the southern part of the country. The Aminu Kano Teaching Hospital is one of the busiest teaching hospitals in Nigeria, located in Kano State, northern Nigeria, which was identified in the first stage of sampling. We then identified the Ahmadu Bello University, Zaria also a busy teaching hospital located in the contiguous state of Kaduna for comparison.

### Health providers

All health providers (consultants, medical doctors in training, nurses, and midwives) working in the obstetrics and gynecology departments and maternity sections of the hospitals were included in the study. Excluded were medical, nursing and midwifery students in training as well as health providers below the level of a skilled birth attendant as defined by the WHO. Also, excluded were all health providers working in the hospitals who were on leave or were absent from the hospitals during the period of the survey.

We first sought permission from the heads of the selected health institutions to conduct the study. Thereafter, we obtained consent from the Heads of Departments of Obstetrics and Gynaecology of the hospitals as well as the Heads of the Maternity Departments. Only the hospitals that provided these three levels of consent were finally included in the study.

Our approach was to interview all qualified health providers (according to the inclusion criteria used) in the hospitals with a structured questionnaire with questions relating to their knowledge and practices of EMOC.

### Questionnaire and method of administration

The questionnaire was organized in four sections. See A8-Knowledge survey for clinical providers in S1 Form. In section one we elicited information about the respondents socio-demographic characteristics–age, sex, educational level, type and level of health professional, number of years of practice, and years since working in the hospital. In section 2 of the questionnaire, we inquired about the availability of policies and protocols relating to the provision of EMOC in the hospitals, including clinical/maternal and perinatal death reviews and surveillance, and membership of safe motherhood related committee or organization. In the third part of the questionnaire, we asked questions about their knowledge of critical EMOC practices and procedures and also about the reported skills in providing the reported services. In the final part of the questionnaire, we solicited information about the confidence in providing the EMOC services and transferring the skills to other health care workers.

The questions were asked in a value-free and mostly in close-end manner. A few questions are open-ended, and were designed to provide subtle information on the answers they provided.

The questionnaires were self-administered, and because all participants were trained in English, they answered in English without a need to translate to the local language. However, a research coordinator was always on stand-by to provide explanation to areas of the questionnaires that were not well understood by the respondent.

### Variables and measures

A measure of knowledge of emergency obstetric complications was generated using responses to ten questions on prevention and treatment of PPH, eclampsia and obstructed labour. The WHO recommendations on methods and drug dosage for the prevention and treatment of these complications [21, 22, 23] were the standards used to classify the responses into correct and incorrect options. An index of knowledge of emergency obstetrics care was created for descriptive and multivariate analyses by aggregating the correct and incorrect responses into a variable with three categories using principal component analysis: low moderate and high.

Competence in clinical skills was measured with response to questions on 18 clinical skills. The providers were asked to state how confident they are in performing the following skills: antenatal care, counseling women on birth preparedness and complications readiness, monitoring labour using partograph, active management of third stage of labour, postpartum care, neonatal resuscitation, caesarean section, vacuum delivery, forceps delivery, laparotomy and repair of ruptured uterus/hysterectomy, use of magnesium sulphate, bimanual compression of the uterus, repair of cervical tears, repair of episiotomy, repair of first and second degree perineal tears, management of postpartum haemorrhage (PPH), and management of eclampsia. The response options were: 1. Confident, I do not need any coaching, 2. Not confident, I need more coaching, 3. I cannot perform this skill, 4. Not applicable (NA)—Not permitted to perform this skill according to country/hospital policy. All NA responses were dropped for the multivariate analysis. The responses were aggregated using principal component analysis to generate a two-category index of competence: low and high (scale reliability coefficient 0.97). High refers to response of confident in 12–18 skills, and low is confident in 0–11 skills. They were also asked whether the skill has been transferred to others (measure of skills transfer). Response options for clinical skill transfer were yes and no. The responses were aggregated using principal component analysis to generate an index of two categories of transfer: low and high (Scale reliability coefficient 0.97). High was yes response in 12–18 skills whereas low was yes in 0–11 skills.

Other variables were facility, professional cadre, sex, age, number of years since qualification, number of years in the current facility, use of partograph in facility, availability of WHO manual for managing complications, frequency of use of the WHO manual, membership of committee or organization related to safe motherhood, existence of clinical/maternal death review, percentage of professional time spent in patient care, clinical training, teaching/educating/instructing and research. Age was collected in single years, but for statistical analysis, it was categorized into three: 23–35, 36–49, and 50–60 years. This categorization was adopted to highlight any inter-generational difference. Years since qualification was categorized into four (1–5, 6–10, 11–20 and 21–35 years), to differentiate among providers who newly qualified, those in their mid-career and older professionals. Number of years spent in the current hospital was categorized into < = 5 years, 6–10, 11–20 and 21–34.

### Analytical strategy

Data analysis was conducted using Stata 12.0 for windows. Description of the study population by selected socio-demographic and professional characteristics for each facility, availability of protocols on EMOC, knowledge of emergency obstetric care, level of competence and skills transfer were presented using absolute numbers and percentages, and median with inter-quartile range. Pearson chi square test was used to determine significance relationship between type of provider (doctors and nurses) and their response to the ten questions on knowledge of prevention and treatment of PPH, eclampsia and obstructed labour, the composite index of knowledge, competence and skills transfer. To determine the factors associated with the reported level of competence and skills transfer by the providers, binary logistic regression model was estimated. A bivariate model was used to select the variables for the multivariate analysis. Variables that were significant in the bivariate analysis at p<0.05 were included in the multivariate analysis. Use of partograph, facility and cadre were not included in this estimation because of the small values in some of the response categories. For instance, for cadre, the response category low was zero in both competence and skills transfer for doctors who were consultants. Estimation for the predictors of knowledge of emergency obstetrics care was conducted with multinomial logistic regression because the outcome variable has three categories (low, moderate and high). Moderate was the base category. All estimation was set at p<0.05 significance level. Variables for the multinomial analysis were selected as described above for the binary logistic regression.

### Ethical approval

Ethical approval for the study was obtained from the World Health Organization and the National Health Research Ethics Committee (NHREC) of Nigeria–number NHREC/01/01/2007–16/07/2014, renewed in 2015 with NHREC 01/01/20047-12/12/2015b. The Chief Medical Directors, Heads of Departments of the hospitals and the participants were informed of the purpose of the study, and verbal consent was obtained from them to conduct the study. They were assured of the confidentiality of information obtained. No names or specific contact information were obtained from study participants.

## Results

### Socio-demographic characteristics and number of respondents per hospital

Nurses and midwives constituted 56.6% of the study population; while medical doctors comprised the remaining 43.4%. Among medical doctors, medical officers and resident doctors (House Officers, Senior House Officers, Registrars and Senior Registrars) accounted for 37.8%, while consultants were 5.6% (Table 1).

**Table 1:** Socio-demographic characteristics and number of respondents per hospitals.

Characteristic	AMHI	ABUTH	AKTH	CHW	CHB	GHM	KGH	SHI	Total
	n = 45(13.2%)	n = 60(17.6%)	n = 37(10.8%)	n = 40(11.7%)	n = 46(13.5%)	n = 60(17.6%)	n = 27(7.9%)	n = 26(7.6%)	n = 341(100%)
**Cadre**									
Nurse/Midwife	40(88.9)	24(40.0)	10(27.0)	21(52.5)	28(60.9)	49(81.7)	21(77.8)	0(0.0)	193(56.6)
Medical Officer	1(2.2)	2(3.3)	0(0.0)	1(2.5)	3(6.5)	7(11.7)	6(22.2)	13(50.0)	33(9.7)
SHO/Registrar	2(4.4)	15(25.0)	8(21.6)	10(25.0)	4(8.7)	3(5.0)	0(0.0)	9(34.6)	51(15.0)
Senior Registrar	2(4.4)	15(25.0)	12(32.4)	5(12.5)	4(8.7)	0(0.0)	0(0.0)	0(0.0)	38(11.1)
Consultant	0(0.0)	3(5.0)	7(18.9)	3(7.5)	4(8.7)	0(0.0)	0(0.0)	2(7.7)	19(5.6)
Others	0(0.0)	1(1.7)	0(0.0)	0(0.0)	3(6.5)	1(1.6)	0(0.0)	2(7.7)	7(2.0)
Not stated	0(0.0)	0(0.0)	0(0.0)	0(0.0)	0(0.0)	0(0.0)	0(0.0)	0(0.0)	0(0.0)
**Sex**									
Male	2(4.4)	17(28.3)	18(48.6)	17(42.5)	15(32.6)	8(13.3)	5(18.5)	18(69.2)	100(29.6)
Female	43(95.6)	42(70.0)	18(48.6)	23(57.5)	31(67.4)	51(85)	22(81.5)	8(30.8)	238(70.4)
Unstated	0(0.0)	1(1.7)	1(2.7)	0(0.0)	0(0.0)	1(1.7)	0(0.0)	0(0.0)	3(0.9)
**Age**									
23–35	15(33.3)	32(53.3)	14(37.8)	17(42.5)	13(28.3)	16(26.7)	11(40.7)	18(69.2)	136(39.9)
36–49	14(31.1)	21(35.0)	18(48.6)	17(42.5)	21(45.7)	22(36.7)	9(33.3)	8(30.8)	130(38.1)
50–60	12(26.7)	4(6.6)	2(5.4)	2(5.0)	10(21.7)	20(33.3)	6(22.2)	0(0.0)	56(16.4)
Unstated	4(8.9)	3(5.0)	3(8.1)	4(10.0)	2(4.3)	2(3.3)	1(3.7)	0(0.0)	19(5.6)
Median(iqr)	39(18)	35(8)	37(5)	36.5(13.5)	42(13)	42(15)	40(15)	33(11)	38(14)
**Number of years since qualification**									
1–5	11(24.4)	11(18.3)	4(10.8)	11(27.5)	7(15.2)	9(15.0)	4(14.8)	10(38.5)	67(19.6)
6–10	8(17.8)	29(48.3)	17(45.9)	11(27.5)	6(13.0)	10(16.7)	5(18.5)	11(42.3)	97(28.4)
11–20	10(22.2)	13(21.7)	9(24.3)	13(32.5)	17(37.0)	19(31.7)	8(29.6)	5(19.2)	94(27.6)
21–35	16(35.6)	7(11.7)	6(16.2)	5(12.5)	16(34.8)	22(36.6)	10(37.0)	0(0.0)	82(24.0)
Unstated	0(0.0)	0(0.0)	1(2.7)	0(0.0)	0(0.0)	0(0.0)	0(0.0)	0(0.0)	1(0.3)
Median(iqr)	13(22)	8(8.5)	10(6)	10(12)	17(15)	18(17)	15(19)	6(4)	12(14)
**Number of years in current hospital**									
<-5	19(42.2)	33(55.0)	20(54.0)	22(55.0)	19(41.3)	15(25.0)	23(85.1)	19(73.1)	170(49.9)
6–10	11(24.4)	17(28.3)	10(27.0)	11(27.5)	13(28.3)	18(30.0)	1(3.7)	6(23.1)	87(25.5)
11–20	12(26.7)	3(5.0)	6(16.2)	4(10.0)	8(17.4)	13(21.7)	1(3.7)	1(3.8)	48(14.1)
21–34	0(0.0)	5(8.3)	0(0.0)	0(0.0)	6(13.0)	14(23.3)	0(0.0)	0(0.0)	25(7.3)
Unstated	3(6.7)	2(3.3)	1(2.7)	3(7.5)	0(0.0)	0(0.0)	2(7.4)	0(0.0)	11(3.2)
Median(iqr)	6(7)	3(4)	5(5)	5(6)	7.5(8)	10(14.5)	1(1)	2.25(5)	5(8)

Note: AMHI—Adeoyo Maternity Hospital Ibadan; ABUTH–Ahmadu Bello University Teaching Hospital, Kaduna; AKTH–Aminu Kano Teaching Hospital, Kano; CHW–Central Hospital Warri; CHB–Central Hospital Benin; GHM–General Hospital Minna; KGH–Karshi General Hospital Abuja; SHI–State Hospital Ijaiye, Abeokuta. iqr–interquartile range.

Across the health facilities, the majority were nurses and midwives except in Ahmadu Bello University Teaching hospitals where 32.4% were Senior Registrars. In general, slightly over 70% of the providers were female. In Adeoyo maternity hospital, Ibadan, only 4.4% were male; but in State hospital Ijaiye, Abeokuta, the majority were male (69.2%). Most of the providers were below age 50 years with a median age of 38 (inter quartile range 14 years). One half of the participants qualified 12 years before the study. Compared to other hospitals, State Hospital Ijaiye had the highest proportion of providers who qualified 1–5 years before the study (38.5%). Most of the providers had spent less than 6 years in the facility except in General Hospital Minna where the majority had worked in the facility for 6 or more years.

### Availability of protocols and policies on EMOC in the hospitals

The use of partograph to monitor labour was reported in all the eight facilities (Table 2). An insignificant number of the providers in AMHI, CHB, GHM, SHI and CHW reported that they do not use partograph or do not know if it is used. Except in AMHI where 64.4% reported that WHO manual for managing complications in pregnancy and childbirth is available in their hospitals, the majority of the respondents reported that the manual is not available or that they do not know. Response to the frequency of use of the WHO manual was inconsistent. For instance, in AMHI, 31% reported daily and 17.8% said rarely. Most of the providers (73%) reported that they do not belong to any organization or committee related to safe motherhood except in GHM where 55% reported membership of such organizations. The least prevalence of providers who reported membership of safe motherhood related organizations or committees is in ABUTH (5%).

**Table 2:** Availability of protocols and policies on EMOC in the hospitals.

Characteristic	AMHI	ABUTH	AKTH	CHW	CHB	GHM	KGH	SHI	Total
	n = 45(%)	n = 60(%)	n = 37(%)	n = 40(%)	n = 46(%)	n = 60(%)	n = 27(%)	n = 26(%)	n = 341(%)
**Use of partograph in facility**									
Yes	41(91.1)	60(100.0)	37(100.0)	39(97.5)	45(97.8)	58(96.6)	27(100)	23(88.5)	330(96.8)
No	3(6.7)	0(0.0)	0(0.0)	0(0.0)	1(2.2)	1(1.7)	0(0.0)	1(3.8)	6(1.6)
Don’t know	0(0.0)	0(0.0)	0(0.0)	1(2.5)	0(0.0)	1(1.7)	0(0.0)	2(7.6)	4(1.2)
Unstated	1(2.2)	0(0.0)	0(0.0)	0(0.0)	0(0.0)	0(0.0)	0(0.0)	0(0.0)	1(0.3)
**Availability of WHO manual for managing complications**									
Yes	29(64.4)	21(35.0)	19(51.4)	11(27.5)	18(39.1)	21(35.0)	10(37.0)	1(3.8)	130(38.1)
No	10(22.2)	16(26.7)	6(16.2)	15(37.5)	19(41.3)	14(23.3)	10(37.0)	13(50.0)	103(30.2)
Don’t know	4(8.9)	20(33.3)	12(32.4)	12(30.0)	8(17.4)	25(41.7)	4(14.8)	12(46.2)	97(28.4)
Unstated	2(4.4)	3(5.0)	0(0.0)	2(5.0)	1(2.2)	0(0.0)	3(11.1)	0(0.0)	11(3.2)
**Frequency of use of the WHO manual**									
Daily	14(31.1)	7(11.6)	7(18.9)	5(12.5)	9(19.6)	16(26.6)	6(22.2)	0(0.0)	64(18.8)
Once a week	3(6.7)	4(6.7)	1(2.7)	1(2.5)	3(6.5)	1(1.7)	1(3.7)	(0(0.0)	14(4.1)
Once a month	1(2.2)	3(5.0)	0(0.0)	0(0.0)	2(4.3)	1(1.7)	0(0.0)	0(0.0)	7(2.1)
Rarely	8(17.8)	4(6.7)	9(24.3)	0(0.0)	4(8.7)	0(0.0)	1(3.7)	1(3.8)	27(7.9)
Never	7(15.6)	3(5.0)	2(5.4)	3(7.5)	3(6.5)	7(11.7)	0(0.0)	0(0.0)	25(7.3)
Not applicable	10(22.2)	36(60.0)	17(45.9)	27(67.5)	24(52.2)	35(58.3)	14(51.9)	25(96.2)	188(55.1)
Unstated	2(4.4)	3(5.0)	1(2.7)	4(10.0)	1(2.2)	0(0.0)	5(18.5)	0(0.0)	16(4.7)
**Membership of committee or organization related to safe motherhood**									
Yes	10(22.2)	3(5.0)	9(24.3)	5(12.5)	4(8.7)	33(55.0)	6(22.2)	2(7.7)	72(21.1)
No	31(68.9)	54(90.0)	26(70.3)	35(87.5)	40(87.0)	20(33.3)	20(74.1)	23(88.5)	249(73.0)
Unstated	4(8.9)	3(5.0)	2(5.4)	0(0.0)	2(4.3)	7(11.7)	1(3.7)	1(3.8)	20(5.9)
**Clinical/maternal death audit in hospital**									
**Yes**	31(68.9)	42(70.0)	27(73.0)	32(80.0)	26(56.5)	50(83.3)	13(48.2)	17(65.4)	238(69.8)
**No**	8(17.8)	8(13.3)	4(10.8)	1(2.5)	12(26.1)	3(5.0)	9(33.3)	7(26.9)	52(15.3)
**Unstated**	6(13.3)	10(16.7)	6(16.2)	7(17.5)	8(17.4)	7(11.7)	5(18.5)	2(7.7)	51(14.9)

Note: AMHI—Adeoyo Maternity Hospital Ibadan; ABUTH–Ahmadu Bello University Teaching Hospital, Kaduna; AKTH–Aminu Kano Teaching Hospital, Kano; CHW–Central Hospital Warri; CHB–Central Hospital Benin; GHM–General Hospital Minna; KGH–Karshi General Hospital Abuja; SHI–State Hospital Ijaiye, Abeokuta.

### Knowledge of emergency obstetric care, competence and transfer of clinical skills

About 64% of the respondents gave the correct answer of “active management of the third stage of labor” as the method for preventing PPH, while 36% provided wrong response to the question (Table 3). Regarding the first line drug they regularly used for the prevention of PPH, 56.9% mentioned misoprostol, while 77.8% mentioned oxytocin. With respect to the dosages of the drugs used for PPH prevention, 41.3% gave the correct dosage of each drug mentioned, while the majority failed to give the correct WHO recommended doses.

**Table 3:** Knowledge of emergency obstetric care (WHO recommended standard), competence and transfer of clinical skills.

Item	All (n = 334)	Doctors (n = 141)	Nurses (n = 193)	Pearson chi2
				p-value
**PPH prevention method**				
Correct	63.8	79.4	52.3	
Incorrect	36.2	20.6	47.7	<0.001
**First line drug for PPH prevention (Misoprostol)**				
Yes	56.9	50.4	61.7	
No	43.1	49.6	38.3	<0.05
**First line drug for PPH prevention (Oxytocin)**				
Yes	77.8	81.6	75.1	
No	22.2	18.4	24.9	>0.05
**Dosage of drug for PPH prevention**				
Correct	41.3	54.6	31.6	
Incorrect	58.7	45.4	68.4	<0.001
**Drug for PPH treatment**				
Correct	76.4	83	71.5	
Incorrect	23.6	17	28.5	<0.05
**Dosage for PPH treatment**				
Correct	20.7	25.5	17.1	
Incorrect	79.3	74.5	82.9	>0.05
**Drug for managing convulsion in eclampsia**				
Correct	85.9	97.9	77.2	
Incorrect	14.1	2.1	22.8	<0.001
**Dosage for convulsion treatment**				
Correct	13.8	12.8	14.5	
Incorrect	86.2	87.2	85.5	>0.05
**Method of preventing obstructed labour**				
Correct	47	64.5	34.2	
Incorrect	53	35.5	65.8	<0.001
**Managing obstructed labour**				
Correct	80.2	89.4	73.6	
Incorrect	19.8	10.6	26.4	<0.001
**Knowledge of emergency obstetrics care Index**				
Low	40.7	22.7	53.9	
Moderate	46.4	63.1	34.2	
High	12.9	14.2	11.9	<0.001
**Competence in clinical skill performance (n = 226)**				
Low	54.4	22.4	88.2	
High	45.6	77.6	11.8	<0.001
**Transfer of clinical skills**				
Low	50.9	20.7	82.7	
High	49.1	79.3	17.3	<0.001

Note: WHO recommendation: Method of preventing PPH: Use of uterotonic drugs oxytocin is strongly recommended. Where oxytocin is not available ergometrine or fixed drug combination of oxytocin and ergometrine or oral misoprostol. Dosage for prevention of PPH: Misoprostol 600ug– 1000ug; Oxytocin 10iu; and Ergometrine 0.5mg. Drug for treatment PPH: 1) Oxytocin 2) Misoprostol 3) Oxytocin + misoprostol 4) Ergometrine. Dosage for treatment of PPH: Oxytocin 10 IU; Misoprostol 800ug– 1000ug; and Ergometrine 0.5mg. Drugs for management of convulsion in patients in eclampsia patients: 1) MgS04 2) MgS0_4_ + Diazepam 3) Diazepam. Dosage for treatment of eclampsia: MgS04—4g; Diazepam 5-10mg. Prevention of obstructed labour: partograph

Management of obstructed labour: Emergency CS/Abdominal delivery

On the drug for treatment of PPH, 76.4% provided correct responses, 20.4% gave the correct answer of oxytocin, being the WHO recommended first line drug treatment of PPH. By contrast, the majority (40.1%) reported the combined use of oxytocin and misoprostol as the first line drug treatment for PPH, while more than 23.0% of the respondents reported misoprostol as the first line drug for treatment of PPH (Breakdown not shown). The majority (79.3%) gave the incorrect dosages for drugs used in the treatment of PPH. With respect to routine drug used for the management of convulsion in a patient with eclampsia, 85.9% correctly mentioned magnesium sulphate (MgS0_4_), while and 14.1% gave other responses. However, the correct dosage of magnesium sulphate for treatment of eclampsia was correctly given by only 13.8% of the respondents.

On method of preventing obstructed labour, 47% gave the correct response which is the active management of labor with parthograph, while 80.2% were correct in their response to the question on the management of obstructed labour.

The relationship between the type of provider and knowledge of prevention and treatment of the three complications were statistically significant except for oxytocin as first line drug for PPH prevention, dosage for treatment of PPH and convulsion. Except in the dosage for convulsion treatment, doctors, were more knowledgeable than the nurses in methods of prevention and treatment of the three complications.

Using the three category index of knowledge generated from the response to questions on prevention and treatment of PPH, eclampsia and obstructed labour, 40.7% of the respondents scored low in knowledge of emergency obstetric care whereas only 12.9% were in the high category. Competence in clinical skills for EMOC measured by self-reported confidence in performing the skills was low for 54.4% of the respondents, and in about half (49.1%), reported transfer of skills to lower level providers was high. Doctors were more likely to score themselves higher in ability to perform and transfer various EMOC skills as compared to nurses and midwives.

### Predictors of competence, transfer of clinical skills and knowledge of EMOC

The results of the multinomial logistic regression presented in Table 4 shows that all cadres of doctors were significantly less likely to express low instead of moderate knowledge of EMOC compared to nurses/midwives. The relative risk of being in the low knowledge category instead of the moderate was 85% (p<0.001) less likely for doctors than nurses/midwives (table not shown).

**Table 4:** Predictors of competence and transfer of clinical skills.

Variable	Competence	Skill Transfer
	OR(95% CI), P	OR(95% CI), P
**Knowledge of emergency obstetrics care**		
Low	1	1
Moderate	4.13(1.34–12.7), <0.05	2.48(0.86–7.09)
High	4.75(1.78–12.6), <0.01	3.31(1.31–8.38), <0.05
**Age**		
23–35	1	1
36–49	1.10(0.32–3.67)	0.67(0.22–2.08)
50–60	0.39(0.03–4.61)	0.23(0.02–2.10)
**Sex**		
Male	1	1
Female	0.08(0.03–0.22), <0.001	0.13(0.05–0.33), <0.001
**Number of years since qualification**	1.01(0.92–1.11)	1.03(0.95–1.12)
**Availability of WHO manual for managing complications**	-	
Yes		1
No		0.99(0.37–2.66)
Don’t know		0.72(0.27–1.93)
**Membership of committee or organization on safe motherhood**		
Yes	1	1
No	0.23(0.07–0.71), <0.05	3.59(1.29–9.97)<0.05
**Mean # of hours spent on patients care per week**	1.00(0.97–1.03)	0.98(0.96–1.01)
**Mean # of hours spent on research per week**	1.11(1.01–1.22), <0.05	1.11(1.02–1.22), <0.05

Note: OR—Odds ratio; CI–Confidence Interval; P–p-value

The risk of low knowledge of EMOC was significantly higher for respondents who were not members of a safe motherhood related body (RRR 3.23 p<0.01) compared to those who were members.

Relative to providers who had low knowledge of emergency obstetric care, those whose knowledge was moderate and high were significantly more likely to report confidence in performing clinical skills (Table 4). Competence varied significantly by sex with females being less likely than males to report confidence in skills performance (OR 0.08 p<0.001). Compared to providers who belong to a committee or organization related to safe motherhood, those who are not members of such organizations were 67% less likely to report confidence in performing clinical skills. Number of hours spent on research per week significantly increased the likelihood of high competence in performance of skills (OR 1.11 p<0.05).

The results of transfer of clinical skills are also presented in Table 5. Relative to providers whose knowledge of EMOC was low, skills transfer was significantly higher for those who had high knowledge (OR 3.31 p<0.05). The odds of reporting that clinical skills have been transferred were significantly less likely among female providers compared to males. High skills transfer was significantly more likely to be reported by providers who are not members a safe motherhood related committee or organization (OR 3.59 p<0.05). Report of high skills transfer increased with higher mean number of hours spent on research per week.

**Table 5:** Multinomial logistic regression predicting the factors associated with knowledge of emergency obstetrics care.

	Low versus moderate	High versus moderate
Variable	RRR(95%CI), P	RRR(95% CI), P
**Cadre**		
Nurse/Midwife	1	1
Medical Officer	0.34(0.12–0.96), <0.05	0.17(0021–1.13)
SHO/Registrar	0.13(0.04–0.40), <0.001	0.25(0.05–1.20)
Senior Registrar	0.09(0.02–0.31), <0.001	0.50(0.13–1.95)
Consultant	0.04(0.00–0.41). <0.01	0.18(0.02–1.26)
**Sex**		
Male	1	1
Female	0.61(0.24–1.57)	1.38(0.10–1.39)
**Availability of WHO manual for managing complications**		
Yes	1	1
No	0.31(0.15–0.62), <0.01	0.87(0.38–1.98)
Don’t know	1.23(0.64–2.35)	0.63(0.22–1.76)
**Membership of committee or organization on safe motherhood**		
Yes	1	1
No	3.23(1.60–6.48), <0.01	0.96(0.41–2.20)

Note: Moderate–base category; RRR–Relative Risk Ratio; CI–Confidence Interval; P–p = value

## Discussion

The study was designed to investigate the knowledge and skills of health providers relating to EMOC practices in eight referral hospitals in Nigeria. We first studied the knowledge and use of standard procedures, guidelines and protocols for the management of obstetric complications and the involvement of the health providers in national and international safe motherhood movements. To assess the knowledge and skills of the health providers, we specifically asked questions about their knowledge and skills in managing primary post-partum haemorrhage, eclampsia and obstructed labour, which are the three leading emergency obstetric complications in Nigeria. We assessed these by asking questions on the availability and use of parthograph, which is a standard procedure recommended by the World Health Organization for the prevention of obstructed labour in developing countries [21], and on the respondents’ knowledge and use of the WHO guidelines for managing complications of pregnancy and childbirth [24]. Although a large proportion of the providers reported that they are aware of, and use parthograph for the management of labour, there was limited evidence to substantiate this. However, only a few of the respondents reported their use of the WHO standard guidelines for management of obstetric complications, and less than a quarter were active in national and international networks relating to the prevention of maternal mortality (safe motherhood). Also, they did not appear to use any specific protocols for the management of the leading pregnancy complications, neither was there evidence of regular reviews of maternal deaths and surveillance in the hospitals.

The lack of protocols for the management of CEOC in the referral hospitals is worrisome. If the hospitals are to accurately perform the recommended signal functions for emergency obstetric care, it is important that health workers should have access to international best practices and procedures relating to EMOC. We believe this can be addressed at the health systems level by ensuring that all facilities providing CEOC are offered the guidelines and trained regularly to use them.

Following the general inquiry regarding EMOC guidelines, we fielded a second set of questions to explore the respondents’ accuracy of knowledge regarding standard practices on the prevention and management of the three leading complications of pregnancy. For the prevention of PPH for which the standard answer based on WHO recommendation [23] would be “active management of the third stage of labour and delivery of the placenta with controlled cord traction”, about 64% of the respondents gave the correct answer. The WHO recommends intravenous administration of 10 international units of oxytocin at the delivery of the anterior shoulder as the first line drug for the prevention of PPH [23]. About 78% of the providers answered this question correctly. By contrast, the question on the first line treatment for PPH with our expected reply being based on the WHO recommendation [23], was correctly answered by 76% and only 21% of the respondents provided the correct dosage.

Similar pattern of responses were associated with the questions relating to the management of eclampsia and obstructed labor. While the WHO recommends the use of Magnesium Sulphate for the management of eclampsia, only about 14% gave the correct dosage of the drug. This suggests that they either do not use the drug or they may be using the drug incorrectly for the management of eclampsia.

Overall, the results using an index for knowledge of EMOC based on the various responses showed that only 13% of the respondents scored high in knowledge of emergency obstetric care whereas 41% were low in knowledge. These results demonstrate the need for in-service training of the providers in delivering EMOC services.

The results of the multinomial logistic regression showed that all categories of medical doctors were more likely to score moderate in knowledge of EMOC as compared to nurses/midwives. The result showing better knowledge of EMOC with doctors than with nurses/midwives is not surprising since doctors have the direct responsibility for managing pregnancy complications [24].

The third and final set of questions we fielded was the respondents’ perceptions and self-reporting of their ability to carry out various clinical procedures relating to EMOC. Since these were self-reporting, it was difficult to confirm the correctness of the responses. However, there is evidence that self-reporting by health providers of ability to conduct clinical procedures may be nearly accurate [25], and therefore, we accord relative reliability to the results obtain from analysis of the question.

The results show high level of self-reporting by resident doctors and consultants of their ability to carry out various obstetric procedures, including antenatal care, delivery care, forceps delivery, caesarean section and manual removal of the placenta. While the nurses and midwives also affirmed that they could carry out the procedures, it is not surprising that a considerably smaller proportion reported the skills as compared to the doctors. With the current focus and recommendations on task-shifting in low income countries [26], it is to be expected that more nurses and midwives should be able to carry out the life-saving procedures relating to EMOC.

One of the functions of health providers working in referring hospitals is to teach junior colleagues and other providers the skills and knowledge to carry out EMOC functions [27]. In this study we investigated this aspect by asking respondents their willingness and ability to transfer the skills to other providers. The results showed that providers with high knowledge of EMOC were three times more likely to report skills transfer as compared to those with low knowledge. Skills transfer was also less likely with female providers as compared to males. Corroborating recommendations in a past study [4], these results underscore the need for intense training of providers in referral facilities on EMOC as this would not only increase their ability to carry out the EMOC functions, but will also enable them to transfer the skills to junior colleagues and mid-level providers.

### Study strengths and limitations

This study has major strengths and also limitations. The strength lies in the multi-centre research design, which allows results to be interpreted at the health systems level. However, since the results obtained did not differ significantly between the eight hospitals studied, we merged the results, which also permitted the use of a larger sample of respondents. The larger sample has allowed a more robust analysis and interpretation of the data. The use of a single hospital would have reduced the sample size and also reduced the external generalizability of the results. The prospective research design also enabled the elicitation of information relating to the current clinical practices of providers and also eliminated confounding due to recall bias.

The major limitation of the study is the use of self-reporting by respondents with no efforts made to substantiate or confirm the information obtained. Thus, it is not known whether the reported skills can actually be carried out by the respondents and the accuracy and correctness with which they are carried out. With procedures such as the use of parthograph and the WHO manual, it is also not known whether the respondents regularly use the protocols, and the accuracy with which they use them. Future research will need to focus on these by conducting specific site assessment and the assessment of the clinical procedures either through direct observations or through exit interviews conducted with beneficiaries of clinical procedures.

Despite these deficiencies, the results of this study provide empirical evidence of the knowledge and skills of health providers in Nigerian referral hospitals with implications for policy change and program implementation.

### Policy and program implications

The results of this study have implications for the training of health providers in referral hospitals in Nigeria. A policy on regular training of staff on EMOC functions need to be put in place in referral facilities in the country. Although a manual exists on EMOC training at the national level [28], there is currently no evidence that this manual is regularly used in the health facilities. A policy to use existing national and international EMOC training manuals and that outlines the periodicity of use should be included in the action plan developed by referral hospitals. This should include efforts to ensure that new information on EMOC practices based on high level evidence are communicated to staff on a regular basis.

Furthermore, clinical protocols on the management of leading complications of pregnancy should be developed and shared with health providers in the health facilities, while reminders should be kept in strategic places in the maternity unit to ensure adherence to the protocol. Also, regular maternal morbidity and mortality reviews should be instituted in the hospitals so that any deviation from the protocols would be immediately identified and corrected.

## Conclusion

The results of this study indicate that the knowledge and reported skills on EMOC practices by health providers in referral facilities in Nigeria is lower than average. We conclude that the in-service training and re-training of health providers should be included in national policy and programs that address maternal mortality prevention in referral facilities in the country.

## Supporting information

S1 Form S1-form A8-knowledge survey for clinical providers 2015. (PDF)

## References

[1] World Health Organization. Trends in Maternal Mortality: 1990 to 2015. Estimates by WHO, UNICEF, UNFPA, The World Bank and the United Nations Population Division. Geneva, Switzerland: World Health Organization; 2015.

[2] Say L, Chou D, Gemmill A, TunÇalp Ö, Moller AB, Daniels JD et al Global causes of maternal death: A WHO systematic Analysis. Lancet Global Health 2014; 2(6):e323–e333. 10.1016/S2214-109X(14)70227-X 25103301

[3] UNDP. A Social Determinant Approach to Maternal Health.: Roles for Development Actors, Discussion paper. Bureau for Development Policy; 2011.

[4] Okonofua F, Randawa A, Ogu R, Agholor K, Okike O, Abdus-salam RA et al Views of senior health personnel about quality of emergency obstetric care: A qualitative study in Nigeria. PLOS ONE 2017; 12(3): e0173414 https://doi/10.1371/journal.pone.0173414 28346519 10.1371/journal.pone.0173414 PMC5367679

[5] WHO, UNFPA, UNICEF and Mailman School of Public Health. Monitoring Emergency Obstetric Care. Geneva, Switzerland: World Health Organization. 2009. ISBN 9789241547734.

[6] National primary health care development agency (NPHCDA): Minimum Standards for Primary Health Care in Nigeria. Abuja, Nigeria: Department of Planning, Research and Statistics, National Primary Health Care Development Agency; 2012. www.nphcda.gov.ng

[7] Geleto A, Chojenta C, Mussa A and Loxton D. Barriers to access and utilization of Emergency Obstetric Care at health facilities in sub Saharan Africa- a systematic Review Protocol. Systematic Reviews 2018; 7(60) 10.1186/s13643-018-0720-y PMC5902829 29661217

[8] Omo-Aghoja LO, Aisien OA, Akuse JT, Bergstrom S, and Okonofua F.E. Maternal Mortality and Emergency Obstetric Care in Benin City, South-South Nigeria. International Journal of Obstetrics and Gynaecology 2013; 1(1):001–006

[9] Onoja-Alexander MOA., Idris SH, Igboanusi CJC, Onoja AD, Istifanus AJ, Olawepo OA. Assessment of the Availability and Accessibility of Emergency Obstetric Care services in Murtala Mohammed Specialist Hospital, Kano, Nigeria. Journal of Community Medicine and Primary Health Care 2017; 29(1)35–43.

[10] Okonofua FE, Sanni Y, Owolabi T, Ekholuenetale M, Kadio B. Unlocking the Benefits of Emergency Obstetric Care in Africa. Africa Journal of Reproductive Health 2016; 20(1);11. ISSN 21313606 10.29063/ajrh2016/v20i1.0 29553173

[11] Okonofua FE, Abejide OR, Makanjuola RO: Maternal mortality in Ile-Ife, Nigeria: A study of risk factors. Studies in Family Planning 1992; 23, 5:319–324. 1475799

[12] Ntiomo L, Okonofua FE, Gana M, Ogu R., Abdus-salam A, Galadanci H et al Prevalence and risk factors for maternal mortality in Nigerian referral hospitals. Int J Women’s Health 2018; 2 1: 10: 69–76. 10.2147/IFWH.S151784 . 29440934 PMC5798564

[13] Okonofua FE, Imosimi D, Igboin B, Adeyemi O, Ekwo C, Orebanjo A, et al Results of Maternal Death Review in Three Hospitals in Lagos State, Nigeria: 2015–2016. Plos One 2017; 12 14; 12 (12): e0188392 10.1371/journal.pone.0188392 29240754 PMC5730145

[14] Kaye DK, Kakaire O, Osinde MO. Systematic review of the magnitude and case fatality ratio for severe maternal morbidity in sub-Saharan Africa between 1995 and 2010. BMC Pregnancy and Childbirth 2011; 11, 65 10.1186/1471-2393-11-65 21955698 PMC3203082

[15] Fikre R. Healthcare provider and the quality of emergency obstetric care in health Centre level in developing countries: a systematic review of the literature. Journal of Health, Medicine and Nursing. 2016; 25, 85–90. ISSNL 2422–8419

[16] Jonas K, Crutzen R, Van den Borne B, Reddy P. Healthcare workers’ behaviours and personal determinants associated with providing adequate sexual and reproductive health services in sub-Saharan Africa: a systematic review. BMC Pregnancy and Child Health 2017; 10.1186/s12884-017-1268-x PMC5348841 28288565

[17] Ebuehi OM, Nkechinyere CG, Muibat SO, Adeyanju SO. Emergency obstetric care: urban versus rural comparison of health workers’ knowledge, attitude and practice in Rivers State, Nigeria–Implications for maternal health care in Rivers State Clinical Medicine & Diagnostics. 2013; 3(2): 29–51.

[18] Ameh CA, Kerr R, Madaj B, Mdegela M, Kana T, Jones S, et al Knowledge and skills of health providers in sub-Saharan Africa and Asia before and after competency-based training in emergency obstetric and early newborn care. Plos One 2016; 11 (12): 10.1371/journal.Pone/0167270. PMC5179026 28005984

[19] Bongban NG, Meyer DJ, Gatunsi PM, Isabella M. Emergency obstetrics knowledge and practical skills retention among hospitals and clinic staff following advanced life support obstetrical training in the Cameroon. Frontiers Women’s Health 2016; 1: 10.15761/FWH.1000110

[20] Ameh CA, van den Broek. Making it happen: training health-care providers in emergency and newborn care. Best Practice and Research Clinical Obstetrics and Gynaecology 2015; 11 29: 8, 1077–1091 10.1016/j.bpobgyn.2015.03.019 25911056

[21] WHO (2008). Managing prolonged and Obstructed Labour: Educational Materials for Teachers of Midwifery (2nd ed.). Geneva, Switzerland: World Health Organization.

[22] World Health Organization. (2011). WHO recommendations for the prevention and treatment of pre-eclampsia and eclampsia. Geneva, Switzerland: World Health Organization 23741776

[23] World Health Organization (2012). WHO recommendations for the prevention and treatment of postpartum haemorrhage. World Health Organization, Geneva. 23586122

[24] WHO. Managing complications in pregnancy and childbirth: a guide for midwives and doctors. 2nd edition, 2017. ISBN 978–92 = 4 = 156549–3. Accessed from www.who.int/maternal-child…/managing-complications-pregnancy-childbirth/en/, on October 1, 2018

[25] Erinosho L. Managing human resource, infrastructure and equipment in Nigeria. In Better Health in Africa. The Nigeria perspective CHESTRE 1996; 18–9

[26] WHO. Task Shifting to tackle Health Workers Shortage- Global Recommendations and Guidelines. Health Systems 2008

[27] Tembo T, Chongwe G, Vwalika B, Sitali L. Signal Functions for Emergency Obstetrics Care as an Intervention for reducing maternal mortality: survey of public and private health facilities in Lusaka District, Zambia. BMC Pregnancy and Childbirth 2017; 17:288 10.1186/S12884-017-1451-6 28877675 PMC5588746

[28] Okaro J.M, Iyoke C.A. The Society of Gynaecology and Obstetrics of Nigeria (SOGON) Plan for Sustainable Reduction in Maternal Mortality: A Review. African Journal of Reproductive Health 2010;14(2): 139–147 21243926

