# Supplementary material for: Assessing the knowledge and skills on emergency obstetric care among health providers: Implications for health systems strengthening in Nigeria
Source: PLoS One. 2019 Apr 8;14(4):e0213719. doi: 10.1371/journal.pone.0213719 (PMC6453439; doi:10.1371/journal.pone.0213719)
Supplement: S1 Form — (PDF) [file pone.0213719.s001.pdf]

RESEARCH TO IMPROVE THE QUALITY OF CARE FOR THE MANAGE-MENT OF OBSTETRICS COMPLICATIONS THAT LEAD TO MATERNAL AND PERINATAL MORTALITY IN NIGERIA

Women's Health and Action Research Centre

Formative Research

FORM A8 – Knowledge and Practice of Emergency Obstetrics Care by Health Providers

*To be filled by all health providers (**Exclude medical, nursing and midwifery students in training**) in Obstetrics & Gynaecology. Your name is not required. Please feel free to write only the truth. Your answers will contribute to a database of information collected to help improve the provi-sion of maternal and newborn health care. All answers are confidential and will not be shared with supervisors or affect your job in any way.*

**001**	Form Number: |__|__||__|__|
**002**	Hospital Name: ____________________
**003**	State: ____________________
**004**	Zone: ____________________
**005**	Date *(DD/MM/YY)*: |__|__| |__|__| |__|__|__|__|

SECTION A:

No	Questions	Response categories			Code
101	Age	[________] years			
102	Sex	1=Male<br>2= Female			[__]
103	What type of health professional are you? (Check only one)	1=Nurse/Midwife<br>2=Medical Officer: ________<br>*Specify Rank*<br>3=SHO/Registrar<br>4=Senior Registrar<br>5=Consultant<br>6=Others: ______________<br>*SPECIFY*			[__]
104	Number of years since qualification			years	
105	How many years have you worked in this hospital?			years	
	**SECTION B**				
106	Please estimate the % of your professional time each week spent in the following activities *(Total should add up to 100%)*				
106a	Patient care				
106b	Clinical training				
106c	Teaching/educating/instructing				
106d	Research				
106e	Other (specify)				
107	Does your institution use the partograph to monitor labour?	1=Yes<br>2=No<br>3=Don't know			[__]
108	Is the WHO manual 'Managing Complications in Pregnancy and Childbirth' available in your institution?	1=Yes<br>2=No<br>3=Don't know			[__]
109	If yes, on average, how many times have you used the manual as reference?	1=Daily<br>2=Once a week<br>3=Once a month<br>4=Rarely<br>5=Never			[__]
110	Do you currently belong to any committees or organizations related to safe motherhood?	1=Yes<br>2=No			[__]
111	If yes, what is (are) the names of the committees) or organization(s)	(1). _ _ _ _ _ _ _ _ _ _ _ _ _ _ _ _ _ _ _<br>(2). _ _ _ _ _ _ _ _ _ _ _ _ _ _ _ _ _ _ _			
112	**SECTION C: As an individual:**				
112a	Approximately how many births do you attend each week?				
112b	Approximately how many antenatal clients do you see each week?				
112c	Approximately how many postpartum clients do you see each week?				

No	Questions	Response categories	Code
113	How would you rate the level of maternal mortality in this hospital	1=High<br>2=Average<br>3=Low<br>4=Don't know	[__]
114	Have you personally attended to women who died during pregnancy?	1=Yes<br>2=No	[__]
115	If yes, approximately how many women in the last one year?		
116	What are the most common conditions that lead to maternal death in this hospital (*please rank them from most to least common)*	(1). ___________________<br>(2). ___________________<br>(3). ___________________<br>(4). ___________________	
117	How common is post-partum hemorrhage (PPH) in your practice?	1=Very common<br>2=Common<br>3=Uncommon/Rare<br>4=Don't know	[__]
118	How many clients with PPH have you managed in the last:	one week	
		one month	
119a	What method do you use to prevent PPH? (please state method)	______________________<br>______________________	
119b	What drug do you most commonly give to women to prevent PPH?	______________________<br>______________________	
119c	What dose do you give?	______________________<br>______________________	
120a	What drug do you use to treat PPH ? (please state method)	______________________<br>______________________	
120b	What drug most commonly do you give to treat PPH?	______________________<br>______________________	
120c	What dose do you give?	______________________<br>______________________	
121a	How common is eclampsia in your clinical practice?	1=Very common<br>2=Common<br>3=Uncommon/Rare<br>4=Don't know	[__]
121b	How many cases of eclampsia did you see in your practice in the last	one week	
		one month	
		9=Don't know	[__]
122a	What drug do you use for the management of convulsion in a patient with eclampsia routinely? (please provide name of drug)	______________________<br>______________________	

No	Questions	Response categories			Code
122b	What dose of the drug do you use?	_ _<br>_ _			
122c	How common is obstructed labour in this hospital?	1=Very common<br>2=Common<br>3=Uncommon/Rare<br>4=Don't know			[__]
123	How many cases of obstructed labour/ruptured uterus did you see in your practice in the last	one week			
		one month			
		9=Don't know			[__]
124	How do you prevent obstructed labour in your practice? *(Name main method)*	1. _<br>9=Don't know			[__]
125	How do you manage obstructed labour in your practice *(Name main method)*	1. _<br>9=Don't know			[__]
126	Do you do clinical/maternal audit in your hospital?	1=Yes<br>2=No			[__]
127	If yes, how often?	1=Weekly<br>2=Monthly<br>3=Quarterly<br>4=Bi-annual			[__]

SECTION D:

Survey of Clinical Skill sets

(Please complete the table below according to the following instructions)

Column A: State the number of cases you have performed in the last one year

Column B: How confident are you in performing the skills? (Please, choose the right option)

1. Confident; I do not need any coaching
2. Not confident; I need more coaching
3. Not confident; I cannot perform this skill
4. N/A Not permitted to perform this skill according to country/hospital policy

Column C: Please check 1 for the skill you have taught to others and 2 if otherwise

SKILL	Column A	Column B	Column C
	**Number of Cases performed in the last 1 year**	**Confidence in performing procedure**	**Whether skill has been taught to others**
	Please, write the number	1=Very confident<br>2=Not very confident<br>3=Not confident<br>4=N/A	1=Yes<br>2=No
Antenatal Care			
Counseling women on birth preparedness and complications readiness			
Monitoring Labor using partograph			
Active management of third stage of labor			
Postpartum care			
Neonatal resuscitation			
Caesarean section			
Vacuum delivery			
Forceps delivery			
Laparotomy and repair of ruptured uterus/Hysterectomy			
Use of magnesium sulphate			
Bimanual compression of the uterus			
Repair of cervical tears			
Repair of episiotomy			
Repair of first and second degree perineal tears			
Management of PPH			
Management of eclampsia			

Thank you.
